# Digital economy: an effective path for promoting residents' health in China

**DOI:** 10.3389/fpubh.2023.1303541

**Published:** 2023-11-22

**Authors:** Xue Zhou, Wen-Ying Yan, Xiu-Ting Li, Han Li, Yi-Zhen Wu, Bao-Chang Xu

**Affiliations:** ^1^School of Economics, Qingdao University, Qingdao, Shandong, China; ^2^School of Business, Xiamen Institute of Technology, Xiamen, Fujian, China

**Keywords:** digital economy, residents' health, green development, CFPS2020, China

## Abstract

The primary prerequisite for socioeconomic growth is good health, hence promoting residents' health is a vital objective of public policies. It is yet up for debate whether or not the digital economy (DE), which will be crucial to future economic growth, will eventually result in improvements in residents' health. Utilizing the China Family Panel Studies (CFPS) data in 2020, we explore how the DE affects residents' health. The findings reveal that residents' health is greatly enhanced by the DE. The eastern region sees a more dramatic improvement in residents' health as a result of the DE. Additionally, the DE can improve residents' health through the promotion of regional green development. The study's findings add to our knowledge of how the DE impacts residents' health while also offering recommendations for achieving universal health.

## 1 Introduction

Health is an important human capital and a major support for socioeconomic development. In spite of the country's continued progress in economic development and social living standards, the residents' health remains not optimistic (1). According to the Report on Nutrition and Chronic Diseases of Chinese Residents (2020), chronic diseases were responsible for 88.5% of Chinese deaths in 2019, and hypertension, hypercholesterolemia, chronic obstructive pulmonary disease, diabetes, and cancer incidence rates increased compared to the statistics recorded in 2015. The Chinese government successively introduced multiple policies aimed at improving residents' health. The “Healthy China 2030” Plan Outline, released in December 2016 by the State Council and the Central Committee of the Communist Party of China, set the target year of 2030 for a considerable improvement in health service capacities. The Healthy China Action (2019–2030), issued in July 2019 by the Healthy China Promotion Committee, advocates for residents to develop a healthy and civilized lifestyle and achieve a healthy life with less illness. In its report to the 20th National Congress of the Communist Party of China, the party stressed the need of “placing the health of individuals in a position of strategic importance of prioritized development and improving policies to promote people's health”. Through the joint efforts of all sectors of society, the level of health literacy among residents across the country steadily improved, from 8.8% before 2011 to 25.4% in 2021.

The DE has recently undergone a period of rapid expansion and has emerged as a new engine of China's growing economy. China's DE ranked second in the world in 2022, with a scale of 50.2 trillion yuan and its proportion of GDP increasing to 41.5%, according to the Internet Information Office's Digital China Development Report (2022). Through constantly upgraded network infrastructure and information tools like smartphones, the DE promotes the penetration of digital technology into deeper areas, with far-reaching implications for economic sustainability and low-carbon growth. Specifically, the DE will be deeply integrated with producing and living activities, promoting the shaping of green consumption concepts, patterns, and production methods, and the creation and growth of smart energy, IoT platforms, and green factories will be encouraged by the DE, which will also greatly empower traditional industries to undertake digital and environmentally friendly transformations. As population health challenges become a more prominent research area in academia (2), can inclusive health dividends result from the DE? Answers to this question are still pending. China is confronted with issues like an aging population and a declining demographic dividend, so it is urgent to develop a talent dividend by enhancing population health in order to capitalize on human resources and support the healthy development of the economy and society. Therefore, scientifically assessing the DE's impact on residents' health is not merely an objective need to comprehensively accelerate digital economic development but also an inevitable requirement to solidly promote the health of all individuals. This study explores the effects of the DE on residents' health and examines its internal mechanisms in an effort to serve as a guide for advancing and improving policies.

## 2 Literature review

The first category of research that has a close connection to this subject is the study of the DE, which mostly researched the DE's influence from two perspectives: macro and micro, covering many fields such as economy, society, and ecology. The DE benefits regional green development, industrial structure upgrading, and green innovation, as has been confirmed by the existing macro-level research (3–5). The analysis of the influence of the DE on enterprise development has been the primary topic of micro-level studies. Implementing digital economic strategies could significantly increase listed Chinese firms' cash holdings, claimed Zhang and Liu (6). Li et al. provided evidence that by easing the constraints of financing, the DE effectively promoted innovation in firms (7). Another type of research is based on a more micro perspective, focusing on the DE's social welfare. Research by Zou et al. found that the DE hampered the sociocultural and psychological integration of migrant workers while facilitating their economic integration (8). Lu et al. argued that digital economics significantly boosted women's employment but failed to enhance women's employment quality in all respects (9). In addition, some studies have focused on DE-relevant issues such as enterprise digitization, automation, information technology, the digital divide, and digital finance (10–14).

The second category of relevant research is the study of residents' health. The majority of currently conducted research has examined how residents' health and medical costs are affected by both macroeconomic issues and personal characteristics. In terms of macroeconomic conditions, Bai et al. pointed out that EPU had a positive spatial spillover effect on China's healthcare expenditure (15). McInerney et al. found that for most of the period from 1994 to 2008, the mortality rate of the older adults was countercyclical, and as the unemployment rate rose, the mental health status of the older adults became worse (16). Ruhm's research suggested that as the economy grew, smoking and obesity increased, while physical activity decreased and diets became less healthy (17). Atalay et al. used Australian residents as a sample and confirmed that rising property prices had a beneficial influence on homeowners' physical health but had an adverse impact on renters' physical and mental health (18). Regarding personal factors, Xu et al. believed that purchasing commercial insurance could improve residents' health (19). Von Dem Knesebeck et al. used data from the 2003 European Social Survey and found that people with a low level of education (middle school or lower) often self-rated their health as poor (20).

Direct studies on how the DE influences residents' health are still lacking. The research that is most pertinent to our study examines how internet development affects residents' health, and it has not yet formed a consensus. One type of research held a positive perspective. Wu et al. discovered that internet development greatly enhanced residents' health (1). Shapira et al. also pointed out that internet use was beneficial for improving the health of older adults (21). Another type of research held the opposite view. According to Zhou et al., internet addiction could reduce children's sleep and exercise time, ultimately damaging their physical and mental health (22). Allcott et al. believed that the intensive use of social media can have an adverse effect on mental health (23).

The impact of the DE and the factors that affect residents' health have generally been thoroughly examined in existing studies from both theoretical and empirical perspectives, and it is generally accepted that the DE plays an advantageous role in promoting regional green development. There are three main aspects of the existing research that need to be further developed and improved: first, the existing literature mostly portrays the development of the DE from the perspective of internet application and development, which is insufficient to accurately reflect the connotation of the DE; second, the existing literature primarily uses inductive reasoning to explore the impact of the DE on residents' health and lacks the support of empirical evidence; and third, additional study is required because the existing literature on the mechanism of the DE on residents' health is not conclusive. Consequently, this study explores the effect and mechanism of the DE on resident' health, aiming to provide directions for China to promote residents' health through digital economic development. The marginal contribution of this study is primarily reflected in three areas: first, it creatively assesses the impact of the DE from the perspective of residents' health, reveals its impact mechanism, and expands and enriches the research in the area of DE and health. Second, the endogenous issues in the research are successfully avoided through instrumental variable regression and other robustness tests. Third, the research findings of this work can serve as recommendations for improving DE policy.

## 3 Research hypotheses

**Figure 1:**
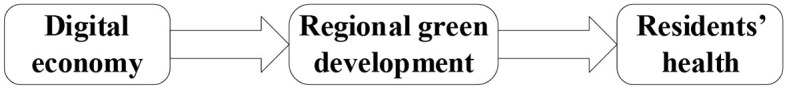
Influence mechanism diagram.

From a realistic standpoint, the construction of China's ecological civilization is fundamentally centered on ecological priority and green development (24), but environmental pollution is posing a growing threat to this development. Environmental pollution is one of the main health risks; thus, improving environmental quality and promoting regional green development is a feasible path to improving residents' health. Theoretically, the DE may help traditional industries make intelligent and sustainable shifts, which will support green production and industries' development (25). Additionally, the DE aids in enhancing energy efficiency (26), lowering resource waste and unneeded environmental emissions, and considerably encouraging the growth of clean energy (27). Overall, the DE successfully promotes regional green development. According to related studies, increases in per capita sulfur dioxide and smoke emissions significantly increased the medical expenses of Chinese residents (28), and carbon emissions had detrimental long-term consequences for residents' health (29). By enhancing environmental quality and improving ecological services, regional green development can address the health demands of the residents. So we speculate that DE may promote green development, thereby enhancing the residents' health. Figure 1 illustrates the fundamental logic behind enhancing residents' health through the DE. Two research hypotheses are put forth in our research.

Hypothesis 1: The DE helps improve residents' health.
Hypothesis 2: The DE may improve residents' health by promoting regional green development.

## 4 Data sources and variables information

### 4.1 Data sources

Data from the CFPS in 2020 is used in this study. The China Social Science Survey Center at Peking University is responsible for carrying out the CFPS, a comprehensive, extensive, and multidisciplinary social tracking survey project. The database created by this project has a wealth of data at the individual, family, and community levels, comprising a wide range of research areas like economic activity, educational accomplishments, family relationships and dynamics, health, and so on. It serves as a reliable source of data samples for this study and is commonly utilized in the field of health economics. In addition, the provincial digital financial inclusion index data as well as the China Statistical Yearbook data, the National Bureau of Statistics data, and the provincial statistical yearbook data are all used in this study. Among these is the provincial digital financial inclusion index, which Ant Financial Group and the Digital Finance Research Center of Peking University jointly established. This index comprises numerous secondary variables in addition to three basic indicators of the coverage, intensity, and level of digital finance. It has been widely used in pertinent studies (30).

### 4.2 Variables information

The level of residents' health, as determined by their self-rated health (19), functions as the dependent variable in this study. Five categories: “unhealthy”, “average”, “relatively healthy”, “very healthy”, and “extremely healthy” are included in the residents' self-assessment of their health condition, given values of 1, 2, 3, 4, and 5, respectively. Self-rated health refers to an extensive assessment of respondents' health that takes into account a number of variables, such as disease severity and health stability. It can accurately depict the residents' health and satisfy the statistical requirements for validity and reliability. The higher the value, the healthier the residents.

The development degree of the DE serves as the study's independent variable. This research assesses the degree of development of the DE from the perspectives of the internet and digital inclusive finance, since the development of the DE relies on the growth of the internet and the promotion of digital finance (31). Specifically, it includes five indicators, namely, the number of internet users per 100 individuals, the proportion of people working in computer services and software, the overall amount of telecommunication services per capita, the number of people using mobile phones per 100 individuals, and the digital financial inclusion index. The entropy value method is used in this study to standardize and shrink the dimensions of the data for the five indicators shown above and build core independent variable DE indicators (9). The primary advantage of the entropy value method is that it is an objective empowerment method that establishes the index weights in accordance with the degree of variation of the index values of various indicators. This effectively avoids the deviation brought on by human factors, strengthens the objectivity of the indicators, and more accurately reflects the degree of the DE's development.

In this paper, the control variables are chosen from three dimensions: individual, family, and province. According to research that has already been conducted, a number of economic and social factors, including education level, per capita income, regional medical investment, and regional economic level, can have a significant impact on residents' health (29, 32). Existing research has also demonstrated that residents' individual characteristics, such as age, gender, marital status, and lifestyle choices like smoking, drinking, and family size, have a considerable impact on health (33). Therefore, in this study, the aforementioned aspects are controlled. To avoid interference from extreme samples, this paper excludes samples with family sizes greater than 10. Continuous variables are then put through a two-tailed 1% level winsorization procedure. The detailed variable definition methods are presented in Table 1.

**Table 1:** Variable definition and assignment.

**Variable**	**Variable assignment**
health	From 1 to 5, get better
dig	The development level of the DE
age	Age (years)
age_2	The square of age
gender	Male = 1, Female = 0
eduyear	Completed years of education
marriage	Married or not, Yes = 1, No = 0
smoke	Smoking or not, Yes = 1, No = 0
drink	Drinking or not, Yes = 1, No = 0
familysize	Number of people living together currently
lnfincomeper	Ln(per capita household income)
lngdp	Ln(provincial GDP)
lnmediexp	Ln(provincial financial expenditure on healthcare)

Married includes four states: being married, cohabiting, divorced, and widowed. Smoking is classified based on whether the respondents have smoked in the past month, and alcohol consumption is classified based on whether the respondents have consumed alcohol more than three times a week in the past month.

Statistics for the primary variables are listed in Table 2. The average for residents' health is 3.09, with a median of 3, indicating that the majority of residents are at the “relatively healthy” level. The DE still has some space for development, since the DE's average only stands at 0.2363, and the difference between the two values, 0.12 for the lowest and 0.91 for the highest, is quite large. Other variables' ranges of values remain within appropriate bounds.

**Table 2:** Descriptive statistics of variables.

**Variable**	**Observation**	**Mean**	**Standard deviation**	**Median**	**Minimum**	**Maximum**
health	20101	3.09	1.20	3	1	5
dig	20101	0.24	0.14	0.17	0.12	0.91
age	20101	45.71	16.68	47	16	95
age_2	20101	2367.31	1570.81	2209	256	9025
gender	20101	0.50	0.50	1	0	1
eduyear	20101	8.87	4.70	9	0	24
marriage	20101	0.83	0.37	1	0	1
smoke	20101	0.27	0.44	0	0	1
drink	20101	0.13	0.33	0	0	1
familysize	20101	4.09	1.85	4	1	10
lnfincomeper	20101	9.86	0.93	9.88	7.31	12.21
lngdp	20101	10.36	0.75	10.49	9.10	11.62
lnmediexp	20101	6.52	0.50	6.57	5.70	7.48

## 5 Model design and baseline regression

### 5.1 Model design

This research creates the following econometric model for analyzing how the DE affects residents' health:


(1) $$\begin{array}{l} health_{ip}=\alpha +\beta dig_{p}+\gamma X_{ip}+\varepsilon_{ip} \end{array}$$

The variables *health* and *dig* stand for residents' health and the provincial DE's level of development, respectively. Individuals and provinces are indicated by the subscripts *i* and *p*, respectively. The control variables are denoted by *X*_*ip*_, and the random error term is indicated by ε_*ip*_. This paper focuses primarily on the coefficient β to reveal how the DE influences residents' health.

### 5.2 Baseline regression

The findings of the baseline regression on how the DE affects residents' health are displayed in Table 3. Only the independent variable is introduced in Column (1), and the independent variable's significant positive regression coefficient shows that the DE greatly promotes residents' health. This conclusion is still confirmed by the estimation findings of the introduction of control variables in Column (2). Based on the aforementioned results, which show the health dividend of the DE, the government can take into account increasing the development of the DE by expanding the application of digital technology and establishing DE policies to encourage the improvement of residents' health.

**Table 3:** Baseline regression.

**Variable**	**(1)**	**(2)**
dig	0.2742^***^	0.4673^***^
	(0.0536)	(0.0646)
age		0.0450^***^
		(0.0035)
age_2		−0.0002^***^
		(0.0000)
gender		−0.1640^***^
		(0.0200)
eduyear		−0.0026
		(0.0022)
marriage		−0.0129
		(0.0310)
smoke		−0.0324
		(0.0222)
drink		−0.1671^***^
		(0.0262)
familysize		−0.0316^***^
		(0.0048)
lnfincomeper		−0.0477^***^
		(0.0104)
lngdp		−0.1530^***^
		(0.0315)
lnmediexp		0.2108^***^
		(0.0451)
*N*	20,101	20,101
*R^2^*	0.0011	0.1157

The ^***^, ^**^, and ^*^ in the table, respectively, show the significance under the significance level of 1, 5, and 10%, and the robust standard errors are shown in the brackets. The same as in the following tables.

This is evident from the control variables' regression results, where the regression coefficients for the variable *age* and its square term, *gender*, *drink*, *familysize*, *lnfincomeper*, *lngdp*, and *lnmediexp*, are found to pass the relevant significance test. The age of residents has a positive influence, but its square term has a negative impact, demonstrating that as people age, their self-rated health level exhibits a reversed “U” trend, originally rising and then gradually falling. The adverse effect of gender shows that men's health level is relatively low, which may be due to men being under greater financial strain than women. Drinking frequently and a large household size worsen residents' health. People who live in households with greater per capita incomes tend to be in worse health, which may be because these families generally have higher living standards and are more likely to have issues like obesity, work pressure, irregular work and rest. The provincial GDP level has an adverse influence, pointing to the fact that residents' health level in economically developed areas is lower. This may be considered because rapid economic development has an adverse effect on the environment by increasing environmental pollution, and the number of diseases brought on by occupational diseases and sub-health is gradually rising. The provincial financial investment in healthcare has a beneficial effect, showing that the more the regional health investment, the healthier the residents are.

### 5.3 Robustness test

This study undertakes robustness testing from three aspects: regression methods, indicator measures, and endogeneity issues, to guarantee the reliability of the study's conclusions.

#### 5.3.1 Change the regression method

Given that the variable *health* in this study has a value of 1–5, which is an ordered categorical variable, order probit and order logit models are used for regression to reduce the estimate error from the OLS method. In addition, the tobit model is further utilized for regression because the dependent variable's range of values is constrained. Table 4's regression results show that the choice of regression methods had no impact on the paper's findings.

**Table 4:** Robustness test 1.

**Variable**	**Order probit**	**Order logit**	**Tobit**
	**(1)**	**(2)**	**(3)**
dig	0.4567^***^	0.7800^***^	0.4673^***^
	(0.0611)	(0.1017)	(0.0646)
age	0.0424^***^	0.0710^***^	0.0450^***^
	(0.0033)	(0.0057)	(0.0035)
age_2	−0.0002^***^	−0.0004^***^	−0.0002^***^
	(0.0000)	(0.0001)	(0.0000)
gender	−0.1583^***^	−0.2731^***^	−0.1640^***^
	(0.0190)	(0.0324)	(0.0199)
eduyear	−0.0012	−0.0056	−0.0026
	(0.0021)	(0.0037)	(0.0022)
marriage	−0.0006	0.0028	−0.0129
	(0.0297)	(0.0496)	(0.0310)
smoke	−0.0288	−0.0517	−0.0324
	(0.0211)	(0.0363)	(0.0222)
drink	−0.1546^***^	−0.2624^***^	−0.1671^***^
	(0.0248)	(0.0433)	(0.0262)
familysize	−0.0302^***^	−0.0513^***^	−0.0316^***^
	(0.0046)	(0.0079)	(0.0048)
lnfincomeper	−0.0435^***^	−0.0752^***^	−0.0477^***^
	(0.0099)	(0.0172)	(0.0104)
lngdp	−0.1514^***^	−0.2458^***^	−0.1530^***^
	(0.0299)	(0.0510)	(0.0315)
lnmediexp	0.2106^***^	0.3739^***^	0.2108^***^
	(0.0428)	(0.0731)	(0.0450)
*N*	20,101	20,101	20,101
*R^2^*	0.0414	0.0429	0.0385

#### 5.3.2 Replace the variable measure

This paper also considers that differences in the measurement of core variables may affect research conclusions, so we change the measurement method of core variables. The variable *health*2 is defined. Give *health*2 a value of 1 for “unhealthy” or “average” self-rated health, a value of 2 for “relatively healthy” and a value of 3 for “very healthy” or “extremely healthy” self-rated health. The binary categorical variable *health*3 is also defined, and a value of 1 is given when the self-rated health status is “very healthy” or “extremely healthy”, and a value of 0 otherwise. The relevant regression findings are listed in Table 5's Columns (1) and (2). The major component approach is additionally applied to create another DE indicator (*dig*2). Column (3) of Table 5 lists the corresponding findings. The aforementioned findings confirm that key variables' measure approach has no influence on the paper's results.

**Table 5:** Robustness test 2.

**Variable**	**(1)**	**(2)**	**(3)**	**(4)**	**(5)**
	**Health2**	**Health3**	**Health**	**Dig**	**Health**
dig	0.3378^***^	0.3665^***^			4.0164^**^
	(0.0412)	(0.0875)			(1.6913)
dig2			1.3972^***^		
			(0.2143)		
officenum1984				0.0002^***^	
				(0.0000)	
age	0.0301^***^	0.0719^***^	0.0449^***^	−0.0006	0.0472^***^
	(0.0022)	(0.0049)	(0.0035)	(0.0004)	(0.0039)
age_2	−0.0002^***^	−0.0005^***^	−0.0002^***^	0.0000^***^	−0.0003^***^
	(0.0000)	(0.0000)	(0.0000)	(0.0000)	(0.0000)
gender	−0.0918^***^	−0.1015^***^	−0.1629^***^	0.0072^***^	−0.1902^***^
	(0.0123)	(0.0266)	(0.0200)	(0.0021)	(0.0247)
eduyear	−0.0039^***^	−0.0325^***^	−0.0028	−0.0006^***^	−0.0004
	(0.0014)	(0.0027)	(0.0022)	(0.0002)	(0.0026)
marriage	0.0036	−0.1304^**^	−0.0138	−0.0180^***^	0.0506
	(0.0195)	(0.0513)	(0.0310)	(0.0037)	(0.0456)
smoke	−0.0181	−0.0611^**^	−0.0329	−0.0033	−0.0213
	(0.0138)	(0.0290)	(0.0222)	(0.0023)	(0.0242)
drink	−0.0823^***^	−0.1415^***^	−0.1685^***^	−0.0148^***^	−0.1126^***^
	(0.0165)	(0.0338)	(0.0262)	(0.0028)	(0.0379)
familysize	−0.0181^***^	−0.0214^***^	−0.0310^***^	0.0040^***^	−0.0460^***^
	(0.0030)	(0.0060)	(0.0048)	(0.0004)	(0.0085)
lnfincomeper	−0.0259^***^	−0.0836^***^	−0.0455^***^	0.0331^***^	−0.1651^***^
	(0.0065)	(0.0129)	(0.0104)	(0.0010)	(0.0570)
Lngdp	−0.0797^***^	−0.0332	−0.1334^***^	0.2230^***^	−0.9501^**^
	(0.0195)	(0.0409)	(0.0308)	(0.0032)	(0.3819)
lnmediexp	0.1294^***^	0.0987^*^	0.1981^***^	−0.2615^***^	1.1570^**^
	(0.0279)	(0.0585)	(0.0450)	(0.0052)	(0.4545)
Kleibergen-Paap rk Wald F statistic					37.252
					[16.38]
Kleibergen-Paap rk LM statistic					64.996
					[0.0000]
*N*	20,101	20,101	20,101	20,098	20,098
*R^2^*	0.1261	0.1252	0.1153	0.3437	−0.0045

For Kleibergen-Paap rk Wald *F*-test, the value in the square bracket is the critical value of 10% level of Stock-Yogo test, and for Kleibergen-Paap rk LM test, the value in the square bracket is the corresponding probability of accepting the original hypothesis.

#### 5.3.3 Addressing endogeneity issues

This study theoretically avoids potential reverse causality issues because the indicators of the DE and residents' health are defined at the provincial and individual levels, respectively. To further exclude interference from endogeneity issues, the instrumental variable in this paper's usage of the instrumental variable approach in regression is set to the historical amount of post offices per million persons (*officenum*1984). We specifically utilized provincial-level corresponding data in 1984 for analysis. The post offices per million persons number was chosen as the instrumental variable because the DE depends highly on regional internet development and earlier internet access depended on the post office system, so it satisfies the requirement for relevance, and it is likely that the number of post offices historically does not have an impact on residents' health, therefore, so it meets the exclusivity requirements. The corresponding estimation results are displayed in Columns (4) and (5) of Table 5. The under identification test and the weak identification test both disprove the initial assumption and support the validity of the instrumental variable. The research conclusion is further confirmed by the fact that the independent variable's regression coefficient in the second stage is obviously positive.

## 6 Further discussion

### 6.1 Heterogeneity test

The sample is then divided into three groups in this study by geographic location: eastern, central, and western (34). The results of subsample regression are then performed, and they are presented in Table 6 as findings. The coefficient of *dig* in the eastern region is obviously positive, which shows that residents' health is greatly promoted by the DE. The central region's regression coefficient of *dig* is not significant, proving that residents' health is barely affected by the local DE. The western region's regression coefficient of *dig* is markedly negative, implying that the DE has a major adverse influence on residents' health. The aforementioned results show that there is regional variability in how the DE affects residents' health. The cause is that the eastern, central, and western regions exhibit substantial differences in the state of the development of the DE, the state of the health infrastructure, and the socioeconomic environment. In comparison to the central and western regions, the eastern region uses digital technology more widely, has a greater variety of digital goods and services, and typically has a higher income and educational level. It is also simpler to access high-quality digital goods and services. As a result, the residents are more significantly impacted by the DE in terms of their health in the eastern region.

**Table 6:** Heterogeneity test—region differences.

**Variable**	**Eastern region**	**Central region**	**Western region**
	**(1)**	**(2)**	**(3)**
dig	0.4855^***^	−0.1030	−1.6318^**^
	(0.0755)	(0.7754)	(0.6532)
age	0.0460^***^	0.0413^***^	0.0468^***^
	(0.0051)	(0.0071)	(0.0064)
age_2	−0.0003^***^	−0.0002^***^	−0.0002^***^
	(0.0001)	(0.0001)	(0.0001)
gender	−0.1993^***^	−0.1143^***^	−0.1693^***^
	(0.0292)	(0.0380)	(0.0393)
eduyear	0.0027	−0.0088^**^	−0.0009
	(0.0036)	(0.0043)	(0.0039)
marriage	−0.0379	−0.0321	0.0364
	(0.0461)	(0.0639)	(0.0557)
smoke	−0.0140	−0.0594	−0.0288
	(0.0332)	(0.0423)	(0.0425)
drink	−0.1886^***^	−0.0861^*^	−0.2432^***^
	(0.0376)	(0.0484)	(0.0571)
familysize	−0.0277^***^	−0.0521^***^	−0.0182^**^
	(0.0074)	(0.0089)	(0.0090)
lnfincomeper	−0.0741^***^	−0.0360^*^	−0.0310
	(0.0160)	(0.0202)	(0.0189)
lngdp	−0.0534	−0.1284	0.1630
	(0.0581)	(0.1552)	(0.1328)
lnmediexp	0.0999	0.2348	−0.1972
	(0.0665)	(0.1980)	(0.1941)
*N*	8,753	5,873	5.475
*R^2^*	0.1135	0.1051	0.1387

### 6.2 Mediation effect test

The following econometric model is developed for the purpose of investigating the mechanism underlying how the DE affects residents' health:


(2) $$\begin{array}{l} health_{ip}=c_{0}+cdig_{p}+e_{ip} \end{array}$$

(3) $$\begin{array}{l} green_{p}=a_{0}+adig_{p}+e_{p} \end{array}$$

(4) $$\begin{array}{l} health_{ip}=b_{0}+c^{\prime }dig_{p}+bgreen_{P}+e_{ip} \end{array}$$

In this model, the mediating variable is regional green development (*green*), which is quantified by the provincial pollution comprehensive index's opposite value. The total provincial wastewater discharge, general industrial solid waste, and sulfur dioxide releases in the waste gas are standardized and reduced in dimensions in this study through the entropy value method to calculate the provincial environmental pollution composite index, whose larger value denotes a more seriously polluted area. Therefore, this study uses the opposite of the provincial environmental pollution composite index to measure regional green development. This study will investigate whether the DE will affect residents' health by influencing regional green development.

In general, there are three steps to testing for mediation effects. The first step focuses primarily on studying the effect of *dig* on *health*, and the major objective of it is to assess the significance of the estimated coefficient *c*. Table 7 displays the corresponding results.

**Table 7:** The relationship between the DE and residents' health.

**Health**	**Coef**	**Std.err**	***t***	***p*>|*t*|**	**[95% conf. interval]**	
dig	0.2742429	0.0535821	5.12	0.000	0.1692176	0.3792682
_cons	2.845985	0.0158089	180.02	0.000	2.814998	2.876972

In light of the results in Table 7, the coefficient *c*is equal to 0.2742 and significantly favorable, revealing that residents' health improves greatly as a consequence of the DE. So that the second step test may be taken out.

The coefficient *a* is what needs to be tested in the second step test, which examines the effect of *dig* on *green*. The pertinent findings are displayed in Table 8.

**Table 8:** The relationship between the DE and green development.

**Green**	**Coef**	**Std.err**	***t***	***p*>|*t*|**	**[95% conf. interval]**	
dig	0.2968942	0.0065145	45.57	0.000	0.2841253	0.3096632
_cons	−0.4312448	0.0019501	−221.14	0.000	−0.4350672	−0.4274224

According to the results in Table 8, regional green development is greatly aided by the DE, with the coefficient *a* equal to 0.2969 and being significantly positive. We therefore get on with the third step test.

The third step test introduces the addition of the mediating variable green development (*green*), primarily testing *c*′and *b*, to explore the association between the DE, green development, and residents' health. Table 9 presents the findings from the regression.

**Table 9:** The relationship between the DE and green development and residents' health.

**Health**	**Coef**	**Std.err**	***t***	***p*>|*t*|**	**[95% conf. interval]**	
dig	0.1911995	0.0565738	3.38	0.001	0.0803101	0.3020888
green	0.2797069	0.0554032	5.05	0.000	0.171112	0.3883018
_cons	2.966607	0.0289706	102.4	0.000	2.909822	3.023392

As can be observed in Table 9, both the coefficients *c*′ and *b* are positively significant and, respectively, equal to 0.1912 and 0.2797. After the introduction of the green development indicator, the benefits of the DE for residents' health do not change, and green development is conducive to enhancing residents' health. The aforementioned findings show that regional green development is an influential way for the DE to benefit residents' health. Overall, the argument is that DE efficiently encourages regional green development by lowering pollution, increasing energy efficiency, and encouraging green industrial transformation, which in turn improves the residents' health.

Based on the tests above, the overall and the direct effect of *dig* on *health* are both significantly positive, respectively, equal to 0.2742 and 0.1912, and that the mediating role of the DE in improving residents' health by encouraging regional green development is significantly positive, equal to 0.0830, accounting for 30.27% of the overall effect.

## 7 Conclusion and insights

This study explores how the DE impacts residents' health by utilizing CFPS data in 2020. According to the study, (1) The DE has a noticeable beneficial effect on residents' health. (2) The function that the DE plays for residents' health differs by region, with the eastern region having the greatest benefit. (3) By encouraging regional green development, the DE enhances residents' health effectively.

The study offers the following three policy insights after considering the results listed above: First, advance digital economic development, and adequate attention should be given to the DE's function in improving residents' health. Specifically, the government should put more emphasis on improving the construction of information infrastructure, enhancing residents' digital literacy, and strengthening the leadership position of the internet, as well as optimizing the regional business environment, supporting the rational allocation of production factors, and creating favorable conditions for the growth of the DE. Make use of the DE's beneficial functions in improving resource utilization efficiency, encouraging green shifts in industries, and promoting regional green development to promote the continual improvement of residents' health.

Second, enhance local environmental pollution inspection and control, assisting the DE in improving residents' health. The government should work to build environmentally friendly industries and support the green transformation of companies in regions with more environmental pressure. Reasonable environmental management policies should be developed based on the carrying capacity of regional resources. In order to remove barriers that stand in the way of regional green development, it is also necessary to strengthen the oversight mechanism for the effectiveness of regional pollution control and the implementation of environmental protection policies, fully ensure the implementation of environmental policies, and impose harsh administrative and monetary penalties on enterprises that violate environmental restrictions. Additionally, to unlock the potential of the DE, we must continue to explore more channels where the DE may benefit residents' health and work with appropriate policies to reinforce the positive benefits of the DE.

Finally, make digital economic development plans that fit local characteristics to drive regional economic development. Considering the regional variations in the DE's function on residents' health, we should work to remove obstacles preventing digital economic development in economically underdeveloped areas and contribute to closing the economic development gap. Specifically, it is essential to integrate regional economic development features with industry comparative advantages, execute distinct regional DE development strategies, and support the growth of digital industries. During this process, full attention should be paid to the actual situation of regional development and people's livelihood issues. The central and western regions should be given fiscal assistance to achieve a steady improvement in the development level of the DE, committed to promoting the application of the DE while driving regional economic development.

These research directions can be explored in the future: First, consider how the DE affects residents' mental health; next, examine how the development of digital infrastructure, digital governance, and digital finance affect residents' health; Third, analyze how certain DE policies affect residents' health.

## Data availability statement

The original contributions presented in the study are included in the article/supplementary material, further inquiries can be directed to the corresponding author.

## Author contributions

XZ: Conceptualization, Methodology, Software, Writing – original draft. W-YY: Formal analysis, Resources, Validation, Writing – original draft. X-TL: Data curation, Project administration, Writing – original draft. HL: Supervision, Validation, Writing – original draft, Writing – review & editing. Y-ZW: Data curation, Funding acquisition, Methodology, Project administration, Resources, Writing – original draft. B-CX: Methodology, Resources, Supervision, Visualization, Writing – review & editing.
